# Enhancing thermoelectric performance by Fermi level tuning and thermal conductivity degradation in (Ge_1−x_Bi_x_)Te crystals

**DOI:** 10.1038/s41598-019-45071-9

**Published:** 2019-06-13

**Authors:** Pai-Chun Wei, Cheng-Xun Cai, Cheng-Rong Hsing, Ching-Ming Wei, Shih-Hsun Yu, Hsin-Jay Wu, Cheng-Lung Chen, Da-Hua Wei, Duc-Long Nguyen, Mitch M. C. Chou, Yang-Yuan Chen

**Affiliations:** 1 Institute of Physics, Academia Sinica, Taipei, Taiwan; 2 Graduate Institute of Manufacturing Technology, National Taipei University of Technology, Taipei, Taiwan; 3 Institute of Atomic and Molecular Science, Academia Sinica, Taipei, Taiwan; 4 Department of Materials and Optoelectronic Science, National Sun Yat-sen University, Kaohsiung, Taiwan; 5 Department of Materials Science and Engineering, National Chiao Tung University, Hsinchu, Taiwan; 6 Computer, Electrical, and Mathematical Sciences and Engineering Division, King Abdullah University of Science and Technology (KAUST), Thuwal, Saudi Arabia

**Keywords:** Materials for energy and catalysis, Thermoelectric devices and materials

## Abstract

In this work, a high thermoelectric figure of merit, *zT* of 1.9 at 740 K is achieved in Ge_1−x_Bi_x_Te crystals through the concurrent of Seebeck coefficient enhancement and thermal conductivity reduction with Bi dopants. The substitution of Bi for Ge not only compensates the superfluous hole carriers in pristine GeTe but also shifts the Fermi level (*E*_F_) to an eligible region. Experimentally, with moderate 6–10% Bi dopants, the carrier concentration is drastically decreased from 8.7 × 10^20^ cm^−3^ to 3–5 × 10^20^ cm^−3^ and the Seebeck coefficient is boosted three times to 75 μVK^−1^. In the meantime, based on the density functional theory (DFT) calculation, the Fermi level *E*_F_ starts to intersect with the pudding mold band at *L* point, where the band effective mass is enhanced. The enhanced Seebeck coefficient effectively compensates the decrease of electrical conductivity and thus successfully maintain the power factor as large as or even superior than that of the pristine GeTe. In addition, the Bi doping significantly reduces both thermal conductivities of carriers and lattices to an extremely low limit of 1.57 W m^−1^K^−1^ at 740 K with 10% Bi dopants, which is an about 63% reduction as compared with that of pristine GeTe. The elevated figure of merit observed in Ge_1−x_Bi_x_Te specimens is therefore realized by synergistically optimizing the power factor and downgrading the thermal conductivity of alloying effect and lattice anharmonicity caused by Bi doping.

## Introduction

Thermoelectric (TE) materials enable the direct energy conversion between heat and electricity that are of great interest in the field of waste heat recovery and solid-state cooling according Seebeck and Peltier effects, respectively. The conversion efficiency is mainly determined by the dimensionless figure of merit *zT* = *σ S*^2^*T*/*κ*, in which *σ*, *S*, *T*,* κ* and *σ S*^2^ are the electrical conductivity, Seebeck coefficient, absolute temperature, thermal conductivity, and power factor (*PF*), respectively. These parameters are strongly coupled with each other, leading to the difficulty in manipulation of *zT* enhancement^1^. To achieve a high *zT* value, band engineering approaches including band convergence^2^, dimensionality reduction^3^, resonant levels^4^, low band effective mass^5^, minority carrier energy filtering^6^, dislocations^7^, 2-dimensional electron gas^8^, have been proposed to improve the performance of electronic contribution, while nanostructuring^9^, multi-scale microstructuring^10^, lattice anharmonicity^11,12^, rattling atoms^13^, liquid phonons^14^, lattice disorder^15^, and interstitial point defects^16,17^ are strategies commonly used for minimizing thermal conductivity of lattice contribution.

To date, group-IV monochalcogenides-based compounds are considered to be the leading TE materials in intermediate temperature range (600–923 K)^18,19^. Among them, GeTe is a heavily *p*-type semiconductor with an inherent high carrier concentration of ~10^21^ cm^−3^. It stabilizes in a non-centrosymmetric rhombohedral structure with an space group *R*3m (No. 160) at room temperature, which undergoes a second-order ferroelectric phase transition to a cubic structure (*F*m $\bar{3}$ m) at 600–700 K, accompanied by an angle distortion of the unit cell from ~57.5° to 60°. The transition temperature depends on the sample stoichiometry and carrier concentration^20^. Its maximum *zT* is close to 1.0 near 700 K. Recently, several pseudobinary system have been found to exhibit *zT* > 1.75 between 600–800 K^21–24^. Besides Sb and Pb, Bi is also a good dopant in this system, i.e., *zT* = 1.3 for Ge_0.94_Bi_0.06_Te melt ingot at 700 K^25^. However, in some cases polycrystalline or single crystals show much better thermoelectric properties as compared to that of the melt ingots. The innovation of this work is that we applied the Bridgman method to grow Ge_1−x_Bi_x_Te crystalline samples, and found an extraordinary high *zT* of 1.9 (700~740 K) in the Bridgman-grown Ge_0.9_Bi_0.1_Te crystal.

## Results and Discussion

**Figure 1:**
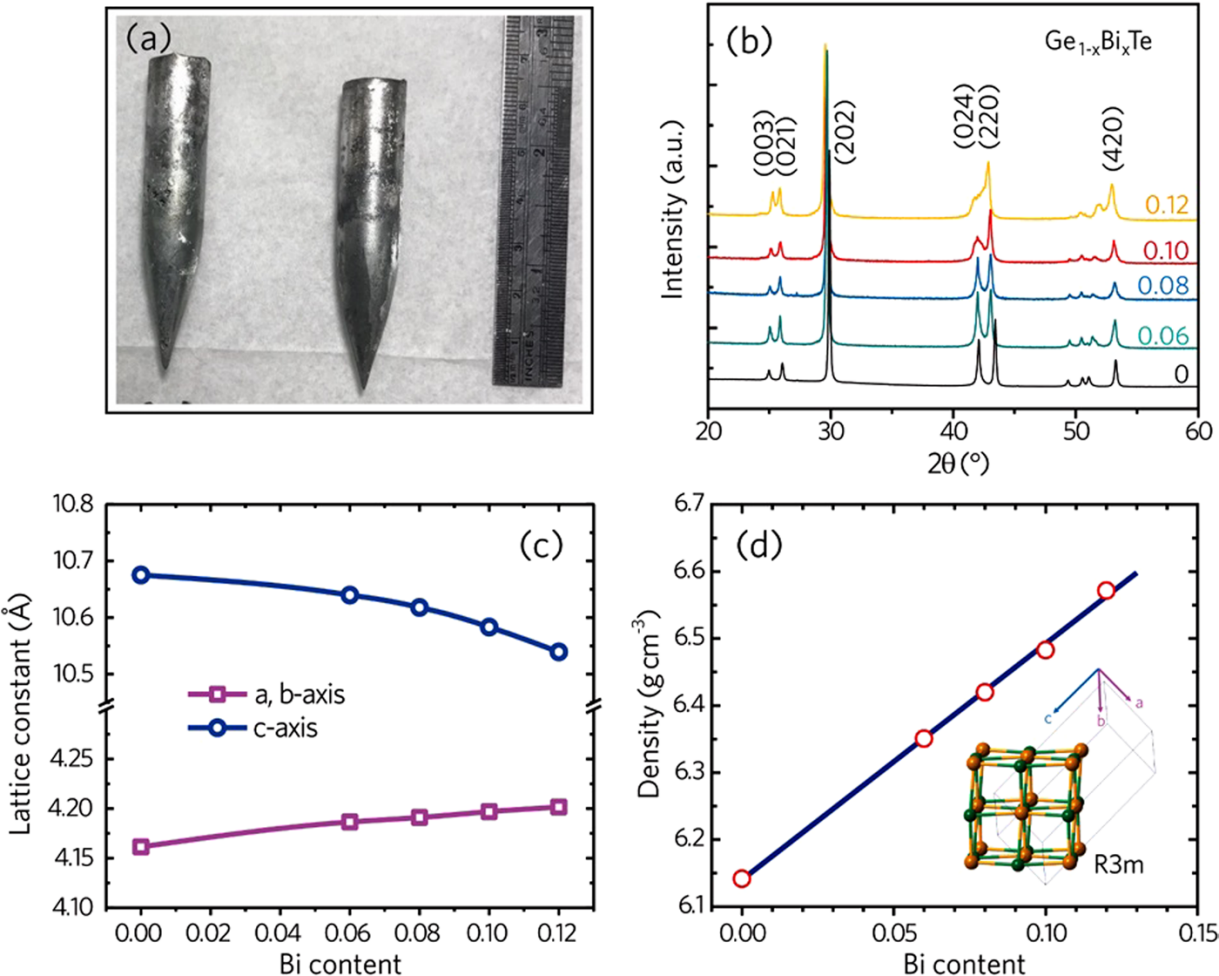
(**a**) Image of representative GeTe crystals. (**b**) XRD patterns of Bi doped Ge_1−*x*_Bi_*x*_Te at 300 K. (**c**) Lattice parameters of all Ge_1−*x*_Bi_*x*_Te crystals. (**d**) Mass density of Ge_1−*x*_Bi_*x*_Te crystals.

The image of as grown Ge_1−x_Bi_*x*_Te ingots are shown in Fig. 1(a). These samples are ~40 g in weight with 13 mm in diameter. They have a rhombohedral lattice at room temperature (the inset in Fig. 1(d)) and are free from secondary phases, as confirmed by the powder X-ray diffraction (XRD) patterns (Fig. 1(b)). With increasing Bi content, the XRD peaks between 42°–44° merge that signifies the increasing cubic nature of Ge_1−x_Bi_*x*_Te structure. From XRD Rietveld refinement, as *x* increases, the lattice parameters of *a* and *b* increase while the lattice parameter *c* decreases monotonically (Fig. 1(c)). Though the ionic radius of Bi is larger than that of Ge, the volume of Ge_1−x_Bi_*x*_Te unit cell is nearly invariant with *x*. This causes the increase of crystal density from ~6.14 g cm^−3^ to 6.58 g cm^−3^, as shown in Fig. 1(d). The thermal stability of samples can also be examined by x-ray diffraction in thermal cycles. Here we carried out the temperature dependence of XRD to check the thermal stability of the Bi doped GeTe samples, for example of Ge_0.9_Bi_0.1_Te (Supplementary Fig. S1), as the sample was heated to 700 K, the diffraction peaks remained the same, thus confirmed the thermal stability of Ge_0.9_Bi_0.1_Te.

**Figure 2:**
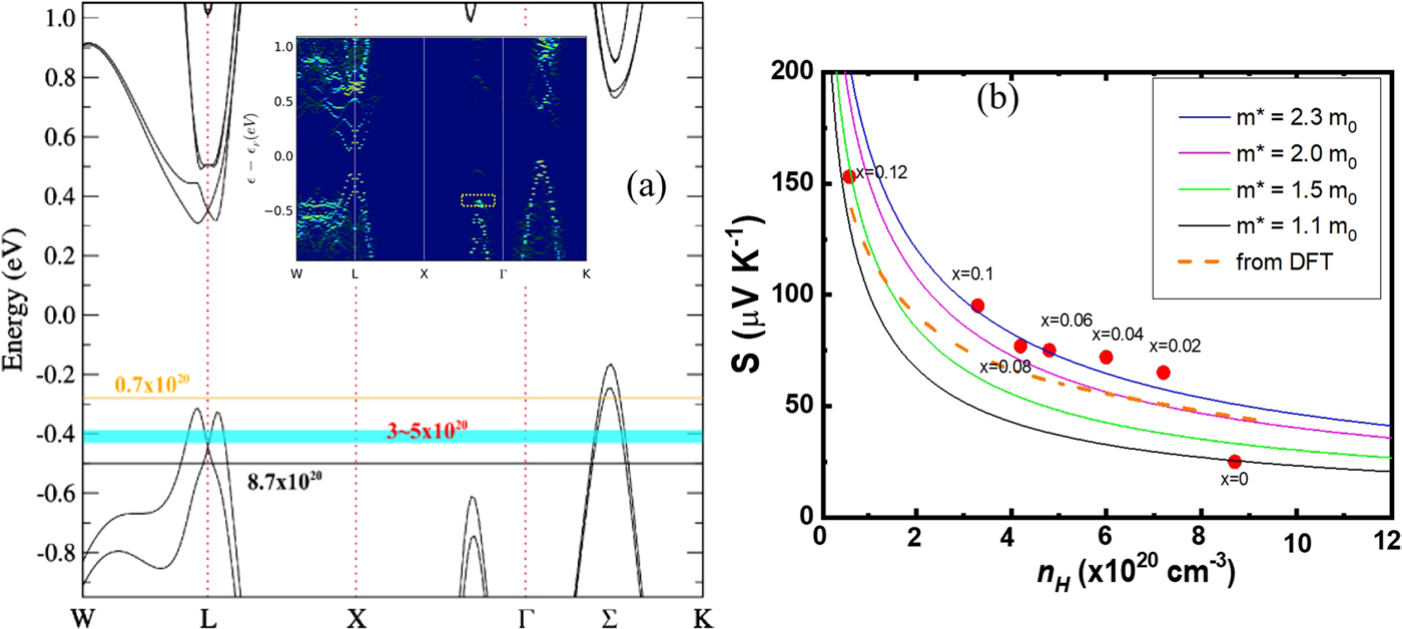
(**a**) DFT calculated Electronic band structure for rhombohedral GeTe with spin-orbital coupling, and the calculated band structure with 7.4% Bi doping shown in the inset figure. (**b**) Seebeck coefficient of Ge_1−*x*_Bi_*x*_Te as a function of carrier concentration at 300 K. The solid lines are derived from the single parabolic band model with the effective mass of 1.1, 1.5, 2.0, and 2.3 *m*_*0*_, respectively. The dashed line is DFT calculated Seebeck coefficient of GeTe as a function of carrier concentration at 300 K.

For pristine GeTe, the inherently high carrier concentration of *n*_*H*_~8.7 × 10^20^ cm^−3^ makes the Fermi level (*E*_F_) falls deeply into the valence band, as shown by the black line in Fig. 2(a), where we show the DFT calculated band structure for the pristine GeTe. The intersections of *E*_F_ with *L* and Σ create hole pockets with large Fermi surfaces that leads to its metallic nature. When the Bi content *x* increases from 0 to 0.12, the *n*_*H*_ reduces from 8.7 × 10^20^ cm^−3^ to 0.7 × 10^20^ cm^−3^ due to the carrier compensation given by the excess electrons from Bi. For a metallic system, Seebeck coefficient at temperature *T* can be described by Mott’s relation^26^:

$$S(T)=\frac{{\pi }^{2}{k}_{B}^{2}T}{3q}{(\frac{1}{N(E)}\frac{dN(E)}{dE})}_{E={E}_{F}},$$

where *k*_B_, *q* and *N*(*E*) are Boltzmann constant, elementary charge, and energy dependent density of state (DOS) near *E*_F_, respectively. Certainly, a large Seebeck coefficient can be brought by a low *N*(*E*) coupled with a steep slope of ∂*N*(*E*)/∂*E* near *E*_*F*_. Note that as the *E*_*F*_ intersects with the “pudding mold valley” at *L* point when *n*_*H*_~3–5 × 10^20^ cm^−3^ (the cyan region in Fig. 2(a))^27–29^, the Seebeck coefficient becomes extremely large and effectively compensates the degradation of electrical conductivity and thus successfully maintains the power factor as large as or even superior than that of the pristine GeTe. The calculated Seebeck coefficients (the orange dashed line in the Fig. 2(b)) were obtained based on the pristine GeTe electronic structure with the BoltzTraP code using rigid-band and constant-relaxation-time approximations^30^. It can be found that the trend of the calculated Seebeck coefficients are consistent with the experimental values. Besides the energy dependent density of state *N(E)*, the effective mass is another factor that has an important influence on the Seebeck coefficient. Hence, the relationship between the carrier concentration and Seebeck coefficient of Ge_1−x_Bi_x_Te was plotted in Fig. 2(b), and the fitting results derived from the single parabolic band model with the effective mass of 1.1, 1.5, 2.0, and 2.3 *m*_*0*_ were presented. Apparently, the effective mass of Bi doped GeTe samples drastically increases from 1.1 *m*_*0*_ to 2.3 *m*_*0*_, partly explaining the enhanced Seebeck coefficient. The DFT calculation with 7.4% Bi doping shows a reduced energy difference between the *L* band and the band in the X-Γ direction (the dashed yellow rectangle in the inset of Fig. 2(a) and Supplementary Fig. S2^31,32^). This will lead to multiple band transport and enhancing the effective mass, which benefits for electrical transport that qualitatively consistent with the experimental result.

**Figure 3:**
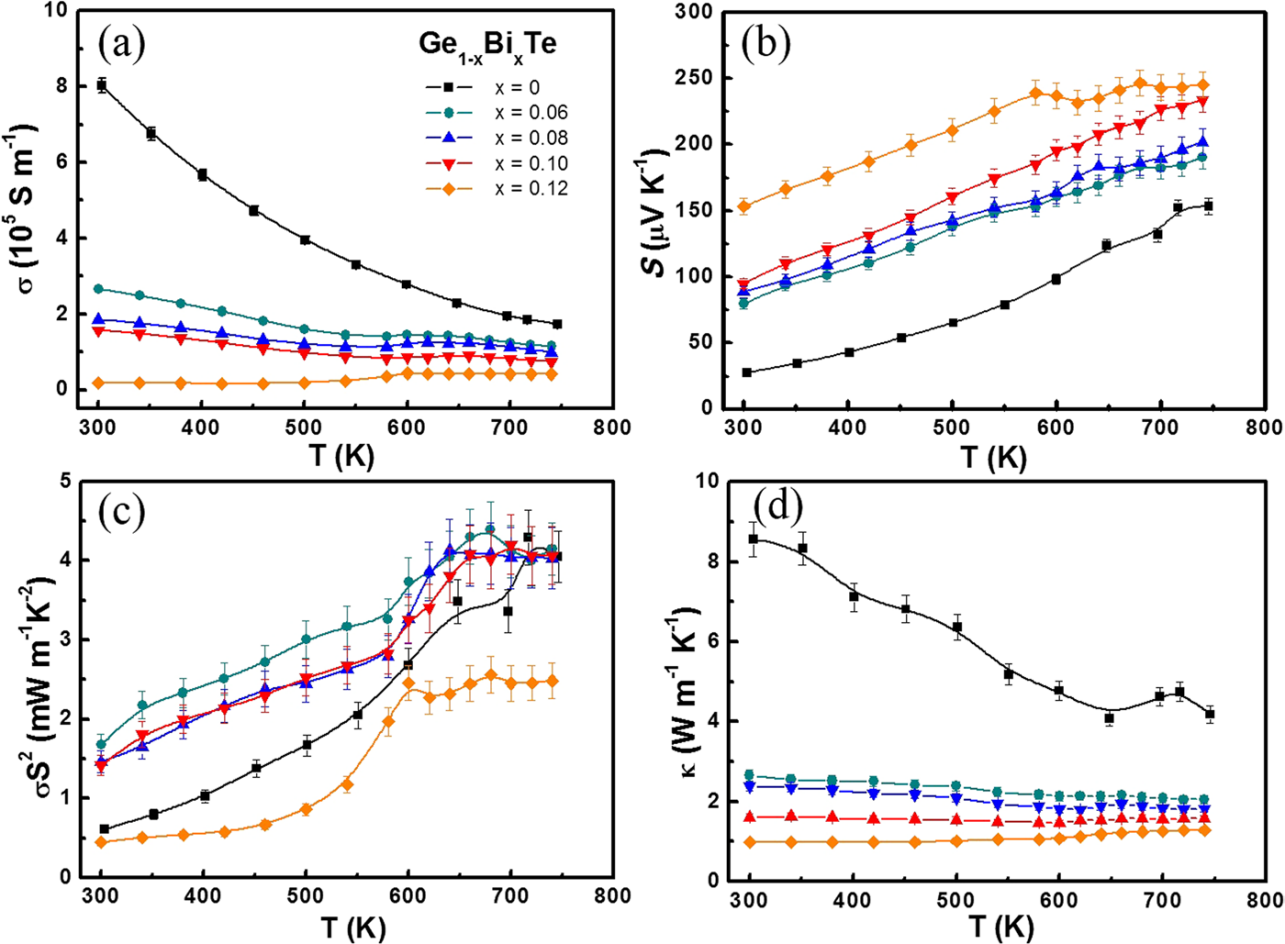
Temperature dependence of (**a**) electrical conductivity *σ*, (**b**) Seebeck coefficient *S*, (**c**) power factor *σS*^2^ and (**d**) thermal conductivity *κ* of all Ge_1−*x*_Bi_*x*_Te samples.

**Figure 4:**
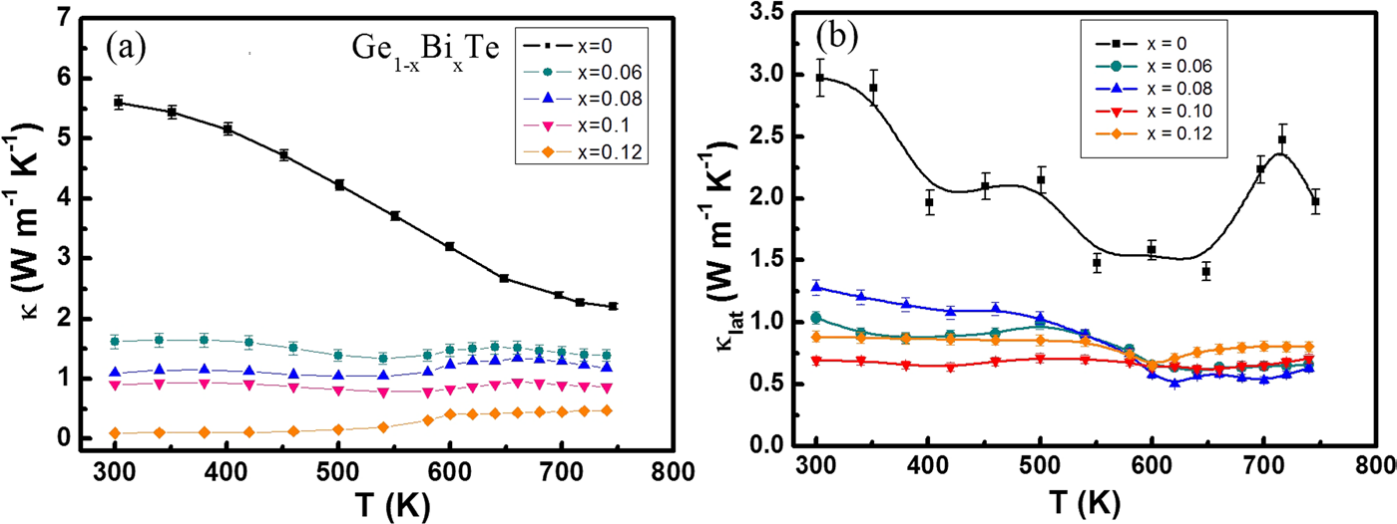
Temperature dependent (**a**) electronic thermal conductivity, κ_e_, and (**b**) lattice thermal conductivity, κ_lat_, of the Ge_1−*x*_Bi_*x*_Te samples.

Figure 3 gives the thermoelectric properties of Ge_1−x_Bi_*x*_Te with *x* = 0–0.12 measured in 300–740 K. The *σ* of all samples decreases with increasing temperature, indicating a degenerated semiconductor behavior (Fig. 3(a)). At room temperature, the *σ* decreases from 8.02 × 10^5^ S m^−1^ to 1.57 × 10^5^ S m^−1^ as *x* increases from 0 to 0.1. As *x* further increases to 0.12, a dramatic reduction of ~97% in *σ* occurs. This can be attributed to the significant reduction in Fermi surface (or DOS) when the *E*_F_ escapes the *L* band. Figure 2(a) shows that the Fermi level *E*_F_ of Ge_0.88_Bi_0.12_Te merely intersects with the Σ band, represented by the orange line, where the DOS (with *n*_*H*_~0.75 × 10^20^ cm^−3^) is greatly reduced. Consequently, only a slight variation in hole concentration will cause a more pronounced *E*_F_ shift. Besides, Bi alloyings do reduce the carrier mobility, and can be presumably attributed to the additional scattering of alloying effects (Supplementary Table S1). This phenomenon is normally seen in similar materials such as SnTe, PbSe, and PbTe. Contrary to the *σ*, the *S* shows an upward tendency with the increasing Bi content, as shown in Fig. 3(b). The *S* of all samples are positive in the whole temperature range, indicating that holes are the dominant charge carriers in this alloying system. In principle, the *S* of all samples increases with temperature increase. For Ge_0.88_Sb_0.12_, a flat *S* plateau is observed as *T* > 550 K, which infers the enhanced bipolar effect arisen from the massive carrier compensation. The point can be further confirmed by the *σ*, which starts to increase as T > 550 K and become nearly invariant for all Bi doped samples, the phenomenon is more obvious for x ≥ 0.06 specimens. It is noticed that the trade-off between the *σ* and *S* can significantly bring down the thermal conductivity of carriers while keeping the high value of *PF* (Fig. 3(c)). Compare to the undoped GeTe, the *PF* of *x* = 0.6, 0.8, 1.0 samples below 600 K are greatly enhanced, while for *x* = 0.12, the temperature-dependent *PF* curve drops dramatically due to the significant reduction in *σ*. The pristine GeTe has a high *κ* of ~8.6 W m^−1^ K^−1^ at room temperature, which decreases with increasing temperature and reaches the minimum value of 4.1 W m^−1^ K^−1^ near phase transition point at ~670 K which is clearly reflected in the discontinuity in *κ* (Fig. 3(d). With the increasing Bi content, the *κ* is substantially reduced. For instance, Ge_0.88_Bi_0.12_Te has the *κ* value of ∼1 W m^−1^ K^−1^ and 1.2 W m^−1^ K^−1^ at 300 K and 740 K respectively, which have about 88% and 71% reduction with respect to that of pristine GeTe. Such substantial reduction in thermal conductivity for Ge_1−x_Bi_x_Te crystals is very helpful in boosting the *zT* value. It is known that the total thermal conductivity κ_tot_ is the sum of the electronic contribution κ_e_ and the lattice contribution κ_lat_. The plot of κ_e_ as a function of temperature for all samples is presented in Fig. 4(a), suggesting that the heat transport of pristine GeTe is mainly (~65%) from carrier contribution. With Bi doping, the κ_e_ was significantly decreased by reducing the p-type carrier concentrations. κ_lat_ is then calculated from κ_tot_ by subtracting κ_e_ (Fig. 4(b)). At 740 K, the κ_lat_ of pristine GeTe is ~1.9 W m^−1^ K^−1^, whereas it is only ~0.7 W m^−1^ K^−1^ for x = 0.1 of Bi, showing a 63% reduction in κ_lat_. κ_lat_ decreases rapidly with increasing Bi doping, which can be attributed not only to the enhanced alloy scatterings but also to the lattice anharmonicity that will be discussed later.

**Figure 5:**
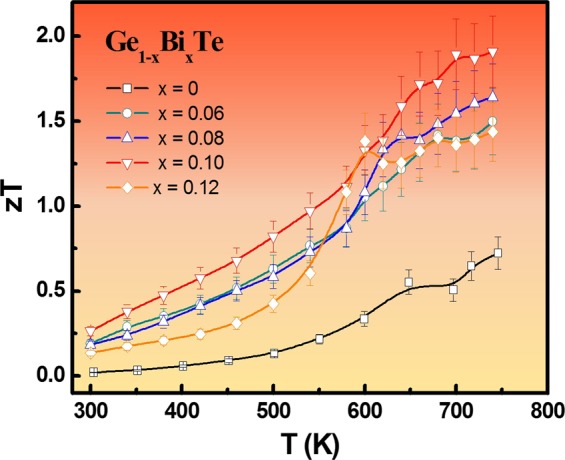
Temperature dependence of figure of merit *zT* of all Ge_1−*x*_Bi_*x*_Te samples.

In addition to the minimized carrier-contributed thermal conductivity and alloying effect, the anomalously low and nearly temperature independent thermal conductivity is also attributed to the large lattice anharmonicity introduced by long pair electrons of Bi cation^33,34^. The valence electronic configuration of Bi is 4f^14^5d^10^6s^2^6p^3^ prefers to form stereochemically active lone pair electrons in many materials. The electrostatic repulsion between the lone pair electrons and the relevant bonding charges have been confirmed to the origin of strong anharmonic phonon-phonon interactions which is able to reduce the lattice thermal conductivity to the nearly amorphous limit. To understand the mechanism of strong anharmonic phonon-phonon interaction of Bi ions in GeTe, further systematical studies are required. Figure 5 shows the temperature dependence of *zT* for all Ge_1−*x*_Bi_*x*_Te specimens. Apparently the *zT* values are significantly boosted by Bi substitution at all temperatures. At highest available temperature *T* = 740 K, the *zT* remarkably achieve the values of 1.5, 1.6, 1.9 and 1.4 for *x* = 0.06, 0.08, 0.1, 0.12 respectively. Especially, a flat plateau of *zT*~1.9 is establish in Ge_0.9_Bi_0.1_Te when >700 K. It is also noted that the largest *zT* value in this paper is as high as 1.9, which is about 46% higher than *zT* = 1.3 reported^25^. The key point is that the elevated *zT* in Ge_1−x_Bi_x_Te specimens prepared by Bridgman method is realized by synergistically optimizing the power factor and downgrading the thermal conductivity of alloying effect and lattice anharmonicity caused by Bi doping. In addition, the thermoelectric data for Ge_0.9_Bi_0.1_Te were repeated for a couple of times, and the narrow statistic distribution of *zT* values clearly confirms the repeatability.

## Conclusions

In summary, the substitution of Ge by Bi in GeTe enables an exquisite manipulation of carrier concentration *n*, Fermi level *E*_F_ and thermal conductivity *κ* to optimum values that raise the *zT* of Bi-doped Ge_1−*x*_Bi_*x*_Te specimens for x = 0–0.12. The combination of theoretical calculations and experimental results elucidates the interrelations between *σ*, *S* and band valleys. Due to the substantial reduction in *κ*, the *zT* of all Bi doped Ge_1−*x*_Bi_*x*_Te specimens are greatly enhanced. A remarkable *zT* of 1.91 at 740 K is achieved in the specimen of Ge_0.9_Bi_0.1_Te, which is comparable to the state-of-the-art high performance thermoelectric material systems.

## Methods

High purity elements of Ge (99.999%), Te (99.999%) and Bi (99.999%) were weighted according to the stoichiometric ratio of Ge_1−*x*_Bi_*x*_Te (x = 0, 0.06, 0.08, 0.10, 0.12), and sealed in evacuated silica tubes. The silica tubes were then heated at 1123 K for 48 hours followed by furnace cooling to room temperature. Ge_1−*x*_Bi_*x*_Te crystals were then grown by using Bridgman method from the pre-melt ingot, with a growth rate of 3 mm h^−1^ at 1123 K. The crystal structure of Ge_1−*x*_Bi_*x*_Te was determined by a PANalytical® X’Pert PRO X-ray diffraction diffractometer (λ = 1.54056 Å). The electrical conductivity and Seebeck coefficient were measured using the ULVAC ® ZEM-3 system. The uncertainty of the Seebeck coefficient and electrical conductivity measurements is about 2~4%. The thermal diffusivity of samples was measured on a NETZSCH LFA 457 laser flash instrument (Supplementary Fig. S3) and the thermal conductivity was calculated from the relationship *κ* = *αC*_p_*d*, where *α* is the thermal diffusivity, *C*_p_ is the heat capacity according to the Dulong-Petit law, and *d* is the mass density, measured by Archimedes’ method. The difference between calculated heat capacity and the measured value is less than 5%, and insure the validity of Dulong-Petit value for the *C*_*p*._ The uncertainty of the thermal conductivity was estimated to be ~5%. Considering the uncertainties for Seebeck coefficient, electrical conductivity and thermal conductivity, the combined uncertainty of *zT* is less than 15%. The carrier concentration was estimated using the relation *p* = 1/*eR*_H_, where *R*_H_ is the Hall coefficient measured by Quantum Design® Physical Properties Measurement System. The uncertainty of the Hall coefficient is ~3%. The first-principles density functional theory (DFT) calculations with spin-orbit coupling effect were performed using projector augmented-wave (PAW) potentials^35–37^, as implemented in the Quantum Espresso package^38^. The exchange-correlation functional was treated by the generalized gradient approximation with the Perdew–Burke–Ernzerhof form^39^. The experimental lattice constants was used with their atomic positions fully relaxed and the kinetic energy cutoff of 750 eV. The Seebeck coefficients were calculated using the BoltzTraP code^30^.

## Supplementary information

Enhancing thermoelectric performance by Fermi level tuning and thermal conductivity degradation in (Ge1-xBix)Te crystals
