# Supplementary material for: Enhancing thermoelectric performance by Fermi level tuning and thermal conductivity degradation in (Ge1−xBix)Te crystals
Source: Sci Rep. 2019 Jun 13;9:8616. doi: 10.1038/s41598-019-45071-9 (PMC6565697; doi:10.1038/s41598-019-45071-9)
Supplement: Supplementary file 1 — Enhancing thermoelectric performance by Fermi level tuning and thermal conductivity degradation in (Ge1-xBix)Te crystals [file 41598_2019_45071_MOESM1_ESM.pdf]

Supplementary Information

Enhancing thermoelectric performance by Fermi level tuning and thermal conductivity degradation in $(Ge_{1-x}Bi_x)Te$ crystals †

Pai-Chun Wei,[1,6] Cheng-Xun Cai,[2] Cheng-Rong Hsing,[3] Ching-Ming Wei,[3] Shih-Hsun Yu,[4] Hsin-Jay Wu,[5] Cheng-Lung Chen,[1] Da-Hua Wei,[2] Duc-Long Nguyen,[3] Mitch M.C. Chou,[4] & Yang-Yuan Chen[1]

[1]Institute of Physics, Academia Sinica, Taipei, Taiwan. [2]Graduate Institute of Manufacturing Technology, National Taipei University of Technology, Taipei, Taiwan. [3]Institute of Atomic and Molecular Science, Academia Sinica, Taipei, Taiwan. [4]Department of Materials and Optoelectronic Science, National Sun Yat-sen University, Kaohsiung, Taiwan. [5]Department of Materials Science and Engineering, National Chiao Tung University, Hsinchu, Taiwan. [6]Computer, Electrical, and Mathematical Sciences and Engineering Division, King Abdullah University of Science and Technology (KAUST), Thuwal, Saudi Arabia. Correspondence and requests for materials should be addressed to Y.Y.C.(cheny2@phys.sinica.edu.tw), C.L.C. (clchen0417@gmail.com) and P.C.W. (pcwei68@gmail.com)

Table S1. The Seebeck coefficient S, carrier concentration n_H, carrier mobility μ_H, and electrical conductivity σ at 300 K for $(Ge_{1-x}Bi_x)Te$ ($0 \leq x \leq 0.12$) samples.

x	S (μV K^{-1})	n_H (x10^{20} cm^{-3})	μ_H (cm^2 V^{-1}.s^{-1})	σ (x10^3 S cm^{-1})
0	25	8.7	57.9	8.02
0.06	75	4.8	32.6	2.50
0.08	77	4.1	28.9	1.85
0.1	95	3.3	27.3	1.45
0.12	153	0.6	19.6	0.19

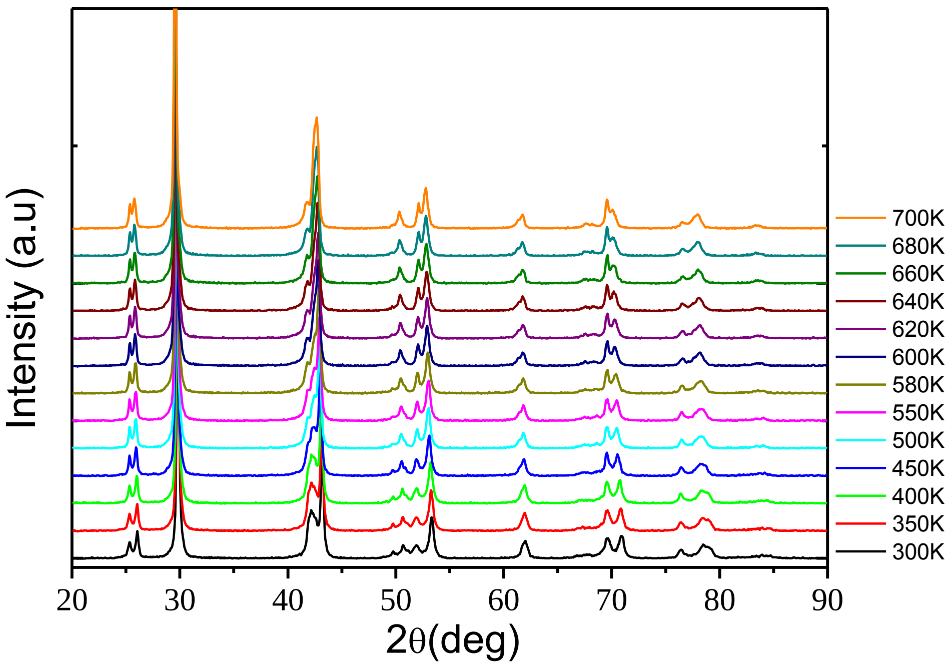

Figure S1. The high-temperature XRD analysis for $Ge_{0.9}Bi_{0.1}Te$

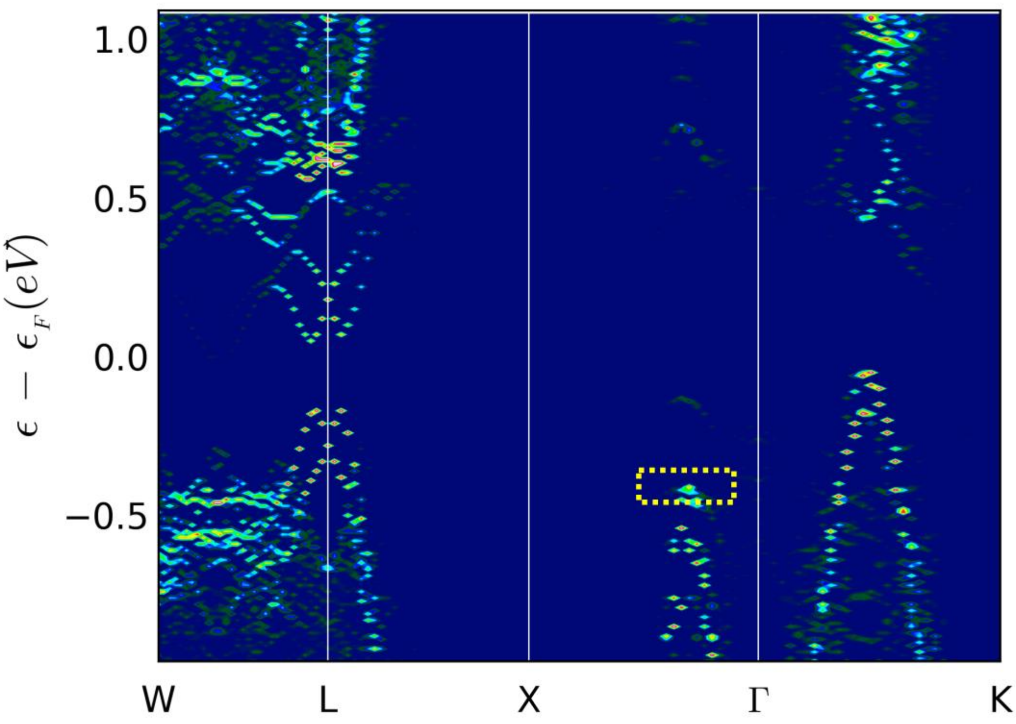

Figure S2. The band structure for the 7.4 % Bi doping is calculated using the band-unfolding technique as implemented in the BandUP code.

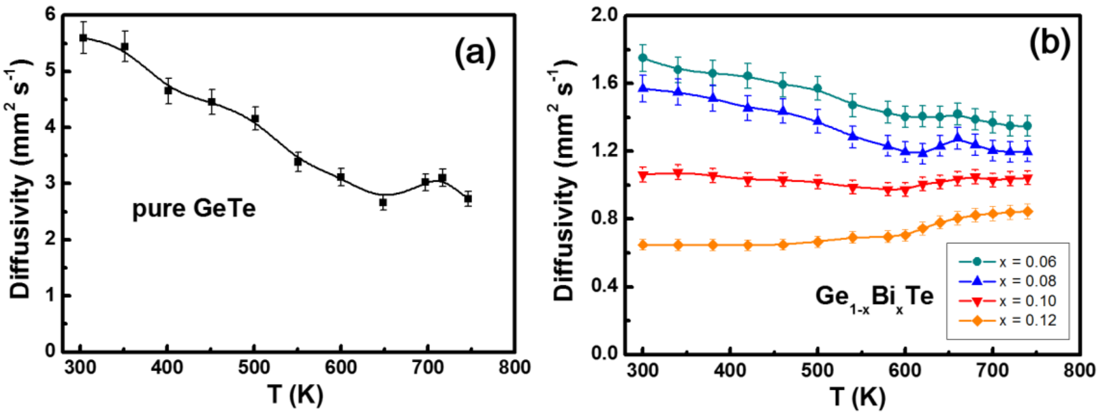

Figure S3. The thermal diffusivity data for (a) pure GeTe, and (b) $Ge_{1-x}Bi_xTe$ samples (x=0.06, 0.08, 0.10, and 0.12).
